# Quantification of Alpha-Gal Expression in Commercial BioProsthetic Heart Valves and Its Potential Mitigation

**DOI:** 10.1016/j.shj.2025.100739

**Published:** 2025-10-13

**Authors:** Andrea Colli, Peter Zilla, Antonio Maria Calafiore, Massimo Padalino, Filippo Naso, Isaac George

**Affiliations:** a Cardiac Surgery Unit, Department of Surgical, Medical and Molecular Pathology and Critical Care, University of Pisa, Pisa, Italy; b Christian Barnard Department of Cardiothoracic Surgery, Groote Schuur Hospital, University of Cape Town, Cape Town, South Africa; c 1st Department of Cardiac Surgery, Henry Dunant Hospital, Athens, Greece; d Department of Cardiothoracic and Vascular Sciences and Public Health, University of Padova, Padova, Italy; e Medical Devices Department, Biocompatibility Innovation S.r.l., Este, Italy; f New York-Presbyterian Hospital, Structural Heart Disease, New York, New York, USA

**Keywords:** αGal, Bioprosthetic heart valve, Glutaraldehyde, Immunogenicity, Polyphenols

## Abstract

**Background and Aims:**

Bioprosthetic heart valves (BHVs) are inherently susceptible to structural degeneration, driven by a combination of mechanical stress, lipid infiltration, glutaraldehyde-induced crosslinking instability, and progressive calcification. Recent evidence has implicated the αGal antigen (galactose-α-1,3-galactose) as an additional contributor to BHV deterioration through activation of innate immune pathways. The present study aims to: 1) perform a quantitative assessment of the residual presence of xenoantigens, specifically αGal, in a range of commercial BHV models; 2) evaluate the efficacy of an experimental polyphenol-based treatment in neutralizing these antigenic determinants; and 3) investigate the long-term stability of glutaraldehyde fixation concerning the potential re-exposure of αGal epitopes.

**Methods:**

Twelve distinct BHV models were subjected to in vitro analysis for αGal antigen quantification both before and following application of an experimental polyphenol treatment. Additionally, glutaraldehyde-fixed bovine pericardial tissues were incubated in a physiologically mimetic, blood-like environment for up to 9 years in real-time to simulate the long-term behavior of BHV materials and assess antigen unmasking associated with glutaraldehyde degradation.

**Results:**

The average count of the αGal epitope in original pericardial valve models was 4.18 ± 0.72 × 10^11^/10 mg of tissue, whereas porcine valve-derived prostheses exhibited a higher mean value of 8.51 ± 2.17 × 10^11^/10 mg. Treatment with the polyphenol formulation resulted in a marked reduction (approximately 99%) in detectable αGal epitopes. Furthermore, glutaraldehyde fixed pericardial tissues subjected to prolonged incubation demonstrated up to 60% re-exposure of previously masked αGal antigens after 9 years, consistent with a progressive compromise of glutaraldehyde crosslinking integrity.

**Conclusion:**

The data confirm that commercially available BHVs retain a substantial immunogenic burden attributable to αGal xenoantigens. Importantly, the overtime degradation of glutaraldehyde crosslinks facilitates the gradual re-exhibition of these epitopes, potentially undermining long-term valve performance. The pronounced efficacy of polyphenol-based treatment in inhibiting αGal antigens highlights its promise as a biocompatibility-enhancing pretreatment strategy for next-generation BHVs.

## Highlights

• For the first time, the residual amount of αGal epitopes has been quantified in the tissue of 12 different commercially available models of BHV.
• The continuous immune stimulation following BHV implantation may be attributed to the depolymerization of glutaraldehyde, which results in the gradual exposure of new antigens. This unmasking effect may contribute to sustained high levels of circulating antibodies, as the immune system encounters newly exposed epitopes over time.
• Polyphenol-based technology has demonstrated significant clinical potential reducing αGal antigen reactivity by up to 90% in multiple BHV models tested.

## Graphical abstract

**Figure:**
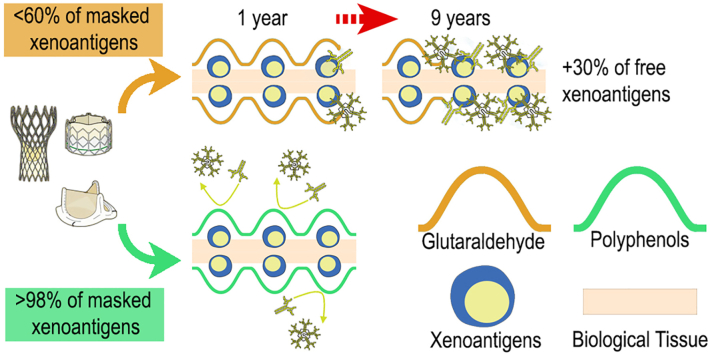

## Introduction

Surgical and transcatheter aortic valve replacements are being performed with increasing frequency, driven by improved clinical outcomes in patients affected by valvular heart disease.^1^ Despite these advancements, bioprosthetic heart valves (BHVs) remain susceptible to structural valve degeneration (SVD), a progressive pathological process that may necessitate reintervention. BHVs are generally manufactured from porcine aortic valve leaflets or bovine/porcine pericardial tissue, both of which are chemically stabilized using glutaraldehyde (GA). The degradation of these biomaterials has been attributed to several interrelated factors, including mechanical fatigue, lipid infiltration, GA crosslinking instability, and pathological calcification.^2^

Recent studies have highlighted a pivotal role for antibody (Ab)-mediated immune responses in the degenerative cascade of BHVs, specifically involving the carbohydrate xenoantigen galactose-α-1,3-galactose (αGal).^3^, ^4^, ^5^, ^6^ This epitope is ubiquitously expressed in most nonprimate mammalian tissues, whereas it is absent in humans and higher primates.^7^^,^^8^ In the human host, continuous microbial stimulation within the gastrointestinal tract induces a lifelong production of anti-αGal Abs, which account for approximately 1%–3% of circulating immunoglobulins and represent the most abundant natural Abs throughout life.^7^

A growing body of experimental, clinical, and translational evidence supports the hypothesis that residual αGal epitopes serve as potent immunological triggers, initiating or amplifying a multifactorial degenerative cascade in BHVs. While early studies primarily focused on immune responses following valve implantation, they collectively underscore the role of αGal-specific immune activation in driving both early and progressive bioprosthetic deterioration. Notably, αGal epitopes have been shown to elicit xenogeneic immune responses, including complement activation and immune cell infiltration,^3^ sustained humoral responses,^4^^,^^5^ and correlations with structural valve failure.^6^ Further investigations have demonstrated that younger patients exhibit heightened susceptibility to αGal-mediated injury due to more robust immune responses^8^ and that αGal remains the most immunodominant xenoantigen implicated in chronic inflammation and tissue degradation.^9^ Clinical cases of early BHV failure in αGal-sensitized individuals^10^ and long-term persistence of anti-αGal Abs^11^ reinforce the pathogenic relevance of this epitope. Moreover, anti-αGal Abs have been consistently detected in explanted, calcified BHVs, even up to 15 years postimplantation in humans, further supporting the immunological relevance of this xenoantigen.^6^ Despite chemical fixation with GA, BHVs continue to elicit an immune response directed against residual αGal antigens.^3^, ^4^, ^5^, ^6^ Importantly, experimental models using αGal-knockout animals have provided direct causal evidence linking αGal to enhanced immune and calcific responses.^12^, ^13^, ^14^ Taken together, these findings support the view that αGal is not a solitary pathogenic agent but a biologically relevant initiator and amplifier of SVD, acting within a broader context of mechanical stress, chemical degradation, and immune activation.^7^

Accordingly, efforts to reduce or eliminate the antigenic footprint of BHVs, especially residual αGal, may offer a promising route to prolong device durability and attenuate immune-mediated structural deterioration.^9^ The present study was designed to expand on these findings by providing a comprehensive quantification of αGal epitopes in a wide array of commercially available BHV models. Additionally, the efficacy of an experimental polyphenol-based treatment in inhibiting the αGal immunogenic potential has been evaluated. Finally, we investigated the long-term behavior of GA-fixed tissues under physiological mimetic conditions to assess progressive antigen unmasking over time.

Although animal-derived tissues used in the fabrication of BHVs express multiple xenoantigens, this investigation focused specifically on αGal, as it is widely acknowledged as the most immunodominant antigen due to its high expression in porcine and bovine tissues.^15^ Moreover, the constitutive presence of circulating anti-αGal Abs in humans (detectable shortly after birth) makes this epitope immunologically significant, as it can provoke an immediate immune reaction without the need for prior sensitization.^3^, ^4^, ^5^, ^6^^,^^9^

In 2013, Naso and colleagues^16^ were the first to attempt a quantitative assessment of the αGal antigen in commercial BHVs, confirming the indication of persistent xenoantigenicity in some of these medical devices. However, such a study suffers from some important limitations, both in terms of the number of BHV models examined and the technical reliability of the early-stage Enzyme-Linked Immunosorbent Assay (ELISA); it could only provide a preliminary indication of αGal persistence in GA-fixed BHVs. The present study builds upon that pioneering work by analyzing a broader and more representative selection of both surgical and transcatheter BHV models, employing a significantly refined ELISA protocol. These methodological advances enable a more accurate, reliable, and reproducible assessment of the residual immunogenic load associated with αGal in commercial BHV tissues.

## Materials and Methods

This study analyzed 2 complementary types of samples. The first consisted of individual leaflets isolated from commercially available BHVs, which were used to quantify αGal antigen expression and to assess the masking efficacy of a polyphenol-based treatment. In addition to GA fixation, these leaflets had already undergone proprietary commercial processing designed to optimize biomechanical properties and to reduce interactions with calcium and coagulation components.

Given that BHVs are primarily composed of GA-fixed bovine pericardium, a second set of bovine pericardial patches fixed in GA under controlled laboratory conditions was employed to investigate the long-term stability of antigen masking conferred by GA alone. Using pericardium without anticalcification treatment as a substrate provided a model without enabled us to isolate the chemical contribution of GA fixation from other variables.

The combination of these 2 sample types enabled a comprehensive assessment of antigen persistence and masking: BHV leaflets offered insight into the performance of treated devices under real-world manufacturing conditions, while GA-fixed pericardial patches provided a controlled model to study the intrinsic behavior of the base material over time. Together, these complementary models enhance both the translational relevance and the mechanistic understanding of our findings.

### Samples Preparation

#### Bioprosthetic Heart Valves Sampling

**Figure 1:**
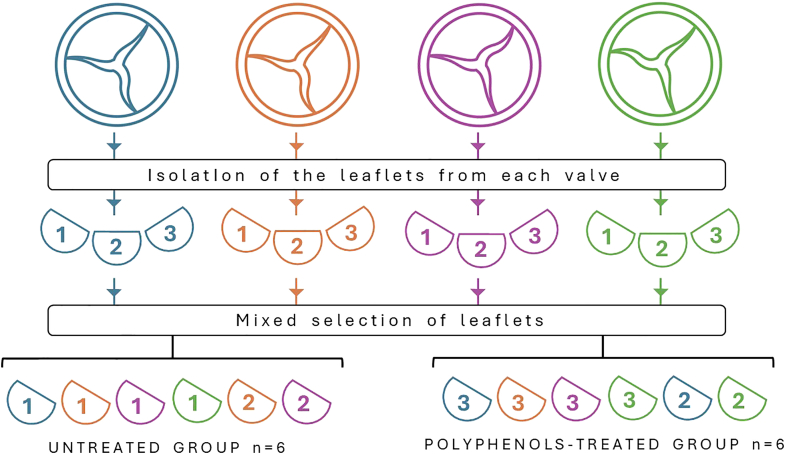
**BHV’s leaflets sampling** To quantify the αGal antigen, 12 different models of bioprosthetic cardiac valves were selected for analysis. For each model, 4 distinct bioprostheses were processed, with each represented by a different color in the figure. The figure also illustrates the sampling and selection scheme, which divided the valve leaflets into 2 groups: the control group (untreated, n = 6) and the test group (polyphenol-treated, n = 6). This methodological approach ensured a high degree of sample heterogeneity and enhanced the statistical robustness of the obtained results. Abbreviations: αGal, galactose-α-1,3-galactose; BHV, bioprosthetic heart valve.

The study involved testing 12 different models of commercially available BHVs. Four bioprostheses for each model were dissected to isolate the leaflets, for a total of n = 12 leaflets/model. It is important to note that each bioprosthetic valve comprises 3 leaflets, typically obtained from pericardial patches derived from different donor animals. Manufacturers intentionally adopt this approach, selecting and combining pericardial tissue from multiple sources to assemble a single valve. This practice is aimed at minimizing the inherent biomechanical variability associated with pericardial tissue, which is known to exhibit anisotropic properties. By incorporating leaflets from diverse donors, this strategy contributes to standardizing the mechanical behavior of the final prosthetic device, thereby enhancing the reliability and reproducibility of experimental findings. Furthermore, the sampling strategy involved collecting individual leaflets from 4 distinct valves of the same commercial model, as illustrated in Figure 1. This methodological approach was designed to enhance the representativeness and reproducibility of the data, thereby increasing the overall accuracy and robustness of the experimental results. All BHVs were taken out of their packaging and washed in sterile phosphate-buffered saline (PBS) at room temperature for 15 minutes (3 times), as per the “Instructions for Use” manual provided by the manufacturer. For each model, n = 6 leaflets were directly analyzed for αGal content. Before quantifying the detectable αGal epitopes, the remaining n = 6 leaflets were treated with polyphenols.

#### Polyphenols-Based Treatment of Commercial BHV Leaflets

An experimental polyphenol-based solution was diluted in PBS at room temperature according to the suggested instructions and followed the previously described methods.^17^, ^18^, ^19^ The formulation is based on a class of polyphenolic compounds, several of which have been tested and shown to confer protective effects on xenogeneic tissues as previously reported in the literature.^17^, ^18^, ^19^ In particular, a first constituent of the polyphenolic mixture is selected from the polyphenols belonging to the class of flavonoids (isoflavones, flavones, flavanols, flavanones, or anthocyanins), and a second one is selected from the phenolic acids or derivatives thereof. The isolated leaflets of commercial BHVs were briefly drained, rinsed with PBS, and then transferred to the freshly set polyphenolic solution. The leaflets were left to react for 2 consecutive 30-minute steps, under constant moderate stirring, in the dark and at room temperature. After the polyphenol treatment, the samples were washed 5 times with isotonic phosphate buffer, each for 15 minutes. The samples thus processed were labeled as “polyphenol-treated,” and αGal epitopes were quantified as previously described.^17^, ^18^, ^19^

#### Bovine Pericardial Patch Preparation

The bovine pericardium was obtained from a certified abattoir Inalca Spa, Castelvetro di Modena, Italy. Since the tissue has been considered a by-product of routine care or industry, there was no need to request authorization for testing. The pericardial samples were harvested from the anterior region of the heart after washing thoroughly in sterile PBS at 4 °C. Thickness and microvascularization were carefully evaluated using an optical viewer and a thickness gauge (Digital Material Thickness Gauge, Electromatic Equipment Co, New York) to select the most homogeneous portions of pericardial tissue. The selected area was mechanically cleaned of adipose tissue debris and dissected. The samples were placed into 12 × 10cm bespoke frames and fixed with GA.

The fixation process was carried out using a GA concentration of 0.6% in PBS, pH 7.4, at room temperature in a dark room.^20^ Subsequently, the GA-fixed pericardial patches were excised in 25 strips measuring 1 × 3cm. To determine the number of αGal epitopes inactivated by GA, a series of pericardial patches (n = 5) were analyzed immediately after the fixation process and defined as T0. This was done to have a reference point to compare the other samples after the incubation times established at 1, 2, 5, and 9 years in real-time (not accelerated). The remaining GA-fixed bovine pericardial strips were placed in a sterile plasma surrogate solution at pH 7.3 (composed of sodium bicarbonate 12.5 mM, sodium carbonate 87.5 mM, and 7% w/v human serum albumin (HSA); the solution was renewed weekly) at 37 °C while being continuously stirred. Protein presence, represented by albumin, is crucial as GA fixation primarily targets tissue proteins such as collagen. Including albumin allowed evaluation of potential chemical interactions or competitive binding effects between plasma proteins and GA-crosslinked tissue. The solution was buffered to maintain physiological pH, critical for GA stability, and samples were continuously agitated to simulate mechanical shear stress from blood flow, which is influenced by plasma viscosity. Additionally, the surrogate was sterilized by filtration to prevent microbial contamination during incubation at 37 °C. At 1, 2, 5, and 9 years, the pericardial strips (n = 5 for each time interval) were processed to quantify the amount of exposed αGal epitopes.^17^, ^18^, ^19^

#### αGal Quantification on BHVs’ Leaflets and GA-Fixed Pericardial Tissue Samples

Tissue samples were gently blotted on filter paper and weighed to express the number of αGal epitopes per 10 mg of dry defatted weight (d.d.w.). Generally, for the αGal quantification, the specimens were then cut into smaller parts and incubated in PBS with (1:50) M86 IgM primary anti-Gal Ab (Enzo, Long Island, New York, USA) for 2 hours at 37 °C with gentle stirring.^17^, ^18^, ^19^ As the ELISA test has already been reported, it will only be briefly described.^21^ A Polysorp 96-well plate was coated with 50 μl of αGal/HSA (5 μg/ml) per well (HSA; Dextra Laboratories, Berkshire, UK) and incubated for 2 hours at 37 °C. After washing 3 times with PBS, 300 μl per well of 1% HSA (Sigma, St. Louis, MO, USA) in PBS was used for blocking (2 hours, RT). The wells were then rinsed 3 times.

A set of wells was loaded with 100 μl of supernatant from samples of polyphenol-treated and untreated BHVs’ leaflets, or GA crosslinked bovine pericardial samples and incubated overnight at 4 °C in darkness. After washing, a secondary HRP-conjugate Ab (1:500) (Dako Cytomation, Glostrup, Denmark) was loaded. Finally, 100 μl of horseradish peroxidase substrate buffer was added to each well for 30 minutes at room temperature in darkness. A plate reader measured the plate absorbance at 450 nm (Sky-scan, Thermo Scientific, Waltham, Massachusetts, USA).

#### Statistical Analysis

Data were analyzed in Microsoft Excel and Prism9 for Windows (v9.0.0, GraphPad Software lnc, San Diego, California, USA) and expressed as mean ± SD. A one-sided unpaired *t*-test assessed significant differences between the polyphenol-treated vs nontreated BHVs at the 0.95 confidence level. A two-sided unpaired *t*-test was used to study the statistical difference between pericardial BHVs vs. Porcine valves BHVs at the 0.95 confidence level. A one-sided paired *t*-test was used for the statistical significance of GA fixation of bovine pericardium patches over time at a confidence level of 0.95.

## Results

### αGal Quantification on Tissue Samples

**Figure 2:**
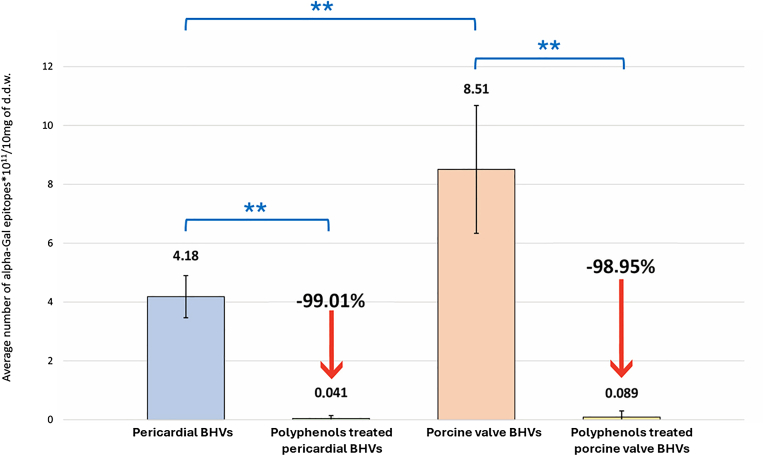
**Average αGal epitopes in bovine and porcine origins BHVs** The mean number of αGal epitopes quantified in leaflets isolated from various commercial BHV models, including those derived from bovine and porcine pericardium (n = 120, both surgical and transcatheter) as well as porcine valve tissue (n = 24). A detailed description of the models used in this analysis is provided in Table 1. Measurements were taken before (blue for pericardial valves and orange for porcine valves) and after polyphenol treatment (yellow for polyphenol-treated valves). Asterisks indicate statistically significant differences (*p* < 0.01). Abbreviations: αGal, galactose-α-1,3-galactose; BHV, bioprosthetic heart valve; d.d.w., dry defatted weight.

The mean number of αGal epitopes found in commercial pericardial BHV models was 4.18 ± 0.72 × 10^11^/10 mg d.d.w. In contrast, porcine valve-derived BHVs (Freestyle 995 and Epic Supra) showed a higher mean value, with 8.51 ± 2.17 × 10^11^/10 mg d.d.w., almost double compared to the pericardial ones (pericardial models vs. porcine valve-derived BHVs, *p* < 0.01, Figure 2).

**Figure 3:**
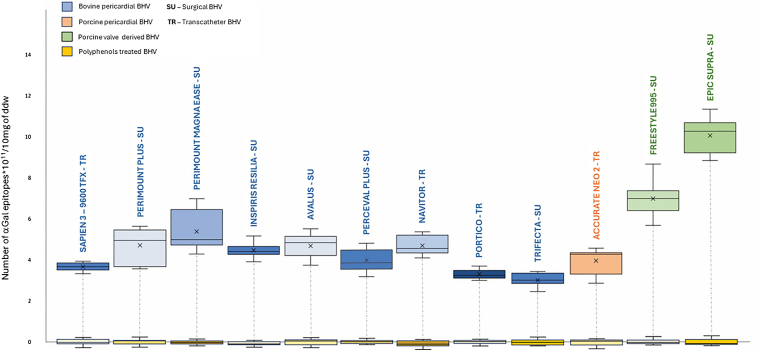
**αGal epitopes quantification in commercial BHVs** Number of αGal epitopes in the original leaflets of 12 different commercial bioprosthetic heart valve models. The models, derived from bovine pericardium (blue), porcine pericardium (orange), and porcine valves (green), are shown. The residual αGal epitopes after polyphenol-based treatment for each model are depicted in yellow (indicating 99% inactivation compared to the original valve models). Box plots display the median (horizontal line), mean (cross inside the box), 25th and 75th percentiles (box edges), and maximum and minimum values (whiskers). Abbreviations: αGal, galactose-α-1,3-galactose; BHV, bioprosthetic heart valve; d.d.w., dry defatted weight; SU, surgical models; TR, transcatheter models.

When examining individual valve models (Figure 3), the bovine pericardial Trifecta GT exhibited the lowest content of αGal antigens: 3.08 ± 0.88 × 10^11^/10 mg of d.d.w. Among the bovine pericardial models, the most recently released valves, Inspiris, Avalus, Navitor, and Portico (Group A), showed similar αGal quantities to the previous generation of SU and TR valves (Perimount Plus and Magna, Sapien 3, and Perceval Plus - Group B), with mean values of 4.27 ± 0.37 × 10^11^/10 mg and 4.15 ± 0.69 × 10^11^/10 mg, respectively (Group A vs. Group B, *p* > 0.05). The porcine pericardial model Accurate Neo 2 has been shown to express an average αGal epitope content of 3.94 ± 0.58 × 10^11^/10 mg, not statistically different from Group A or B (*p* > 0.05). Differently, the old-generation porcine-derived models (Epic Supra and Freestyle 995) showed a statistically higher epitope content than all their counterparts (Group A and B, Accurate Neo2, *p* < 0.01 for each comparison group), reaching 8.51 ± 2.17 × 10^11^/10 mg. The number of αGal epitopes for each specific BHV model has also been reported in Table 1.

**Table 1:** List of commercial models of BHVs with several details among which is the relative amount of αGal

Model	Year of FDA approval∗	Manufacturer	Tissue origin	Valve type	N of αGal epitopes ×10^11^/10 mg in commercial tissue
Navitor	2020	Abbott	Bovine per.	Aortic TR	4.68 ± 0.42
Accurate neo 2	2020	Boston Scien.	Porcine per.	Aortic TR	3.94 ± 0.58
Inspiris Resilia	2018	Edwards Lifesc.	Bovine per.	Aortic SU	4.45 ± 0.33
Avalus	2017	Medtronic	Bovine per.	Aortic SU	4.67 ± 0.52
Portico	2017	Abbott	Bovine per.	Aortic TR	3.28 ± 0.21
Perceval plus	2016	Corcym	Bovine per.	Aortic SU	4.00 ± 0.68
Trifecta GT	2016	Abbott	Bovine per.	Aortic SU	3.08 ± 0.88
Sapien 3	2015	Edwards Lifesc.	Bovine per.	Aortic TR	3.65 ± 0.19
Perim. Magna Ease	2010	Edwards Lifesc.	Bovine per.	Aortic SU	5.36 ± 0.89
Epic supra	2008	Abbott	Porcine valve	Aortic SU	10.04 ± 0.78
Freestyle 995	2001	Medtronic	Porcine valve	Aortic SU	6.97 ± 0.84
Perim. Plus	1983	Edwards Lifesc.	Bovine per.	Aortic SU	4.69 ± 0.82

Abbreviations: αGal, galactose-α-1,3-galactose; BHV, bioprosthetic heart valve; FDA, Food and Drug Administration; GT, glide technology; SU, surgical valve; PER pericardium; TR, transcatheter valve.

∗ The year of FDA approval was obtained from https://www.fda.gov/.

### αGal Mitigation After Polyphenols-Based Treatment

Polyphenol-based treatment has effectively decreased residual immunogenicity in all treated commercial BHV models, as shown in Figure 2 (yellow bar). The treatment significantly inhibits the free-to-react αGal epitopes, with an average reduction of 99.01%. The yellow bars in Figure 3 provide individual values for each BHV after the polyphenol-based treatment.

### Shielding Effectiveness of GA

**Figure 4:**
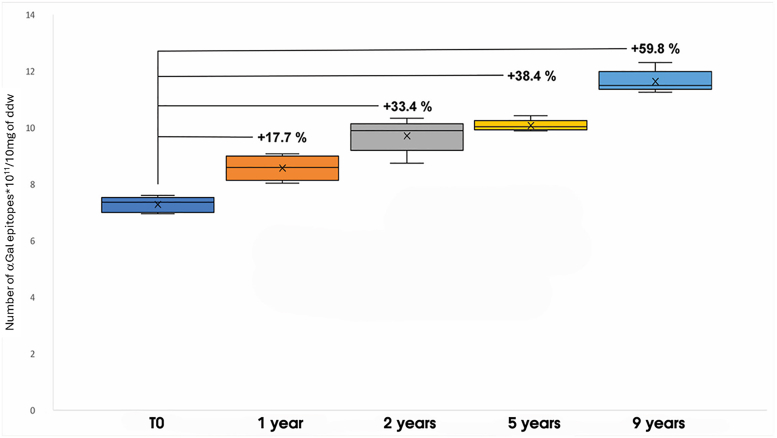
**Unmasking of αGal epitopes** Quantification of αGal epitopes in GA-fixed bovine pericardial samples. The quantification was performed after incubating the samples for specific durations in a sterile blood surrogate solution. The graph shows the percentage increase in antigenic exposure over time, compared to patches analyzed at T0 (immediately after GA fixation). Statistically significant differences are noted as follows: 1 year, 2 years, 5 years, and 9 years vs. T0 (*p* < 0.05); 1 year vs. 2 years (*p* < 0.05); 2 years vs. 5 years (*p* > 0.05). Abbreviations: αGal, galactose-α-1,3-galactose; d.d.w., dry defatted weight; GA, glutaraldehyde.

Incubating GA-fixed pericardial strips in a sterile plasma surrogate solution at 37 °C with moderate mechanical agitation led to increasing exposure of αGal epitopes over time. The percentages of epitopes exposed in the various years (1, 2, 5, and 9) were compared to the αGal epitopes quantified in pericardial samples immediately after the GA fixation process (T0). After 9 years of real-time *in vitro* stimulation, the tissue showed a 59.8% increase in newly exposed αGal antigens (T0 vs. 9 years, p < 0.01, Figure 4). The study found that 17.7% of previously masked αGal epitopes were exposed after 1 year of GA fixation, 33.4% after 2 years, and 38.4% after 5 years (T0 vs. 1 year, T0 vs. 2 years, T0 vs. 5 years, and T0 vs. 9 years, p < 0.01, Figure 4). There was no statistically significant difference in the values between the 2 and 5 years (*p* > 0.05).

## Discussion

The classical degeneration of BHVs is initiated by a multifactorial cascade involving mechanical stress, biochemical degradation, and immune-mediated injury. Early pathological events include platelet adhesion and activation, complement system engagement, and macrophage infiltration, which collectively promote local inflammation, fibrotic remodeling, and progressive calcification of the valve leaflets.^22^^,^^23^ A critical contributing factor is the chemical instability of GA, the standard fixative used in BHV manufacturing. Over time, GA can depolymerize, exposing calcium-binding sites and failing to fully mask antigenic determinants, thereby facilitating mineral deposition and immune recognition.^22^^,^^24^ These processes compromise the structural integrity and function of the valve, often necessitating reintervention. However, the consistent observation of immune activation suggests that degeneration is not solely a mechanical or chemical phenomenon. The presence of specific Abs, such as anti-αGal, anti-N-glycolylneuraminic acid, and anti-nonhuman collagen, indicates that the host immune system recognizes the implant as foreign, implicating adaptive immunity alongside innate mechanisms.^3^, ^4^, ^5^, ^6^

In recent years, significant progress has been made in elucidating the immunological mechanisms activated by the implantation of BHVs,^25^^,^^26^ making evident that an immune process may be involved in the initial stage of SVD in humans, supported by specific Abs against various xenoantigens expressed by the BHV tissue. Although the contribution of immune mechanisms to BHV deterioration remains a topic of ongoing debate, both experimental^22^^,^^27^ and clinical^25^^,^^28^ studies have consistently supported this hypothesis since the mid-1980s. The relationship between tissue-specific immune reactivity to αGal and the occurrence of dystrophic calcification, inflammation, and leaflet tearing in BHVs in vivo is increasingly suggested.^6^^,^^29^ The literature indicates that following BHV implantation, circulating levels of anti-αGal Abs rise significantly, with detectable increases observed as early as 10 days postimplantation. Peak concentrations of IgM and IgG isotypes are typically reached within 3 months.^3^, ^4^, ^5^ Beyond the humoral response, BHV implantation also activates the classical complement cascade, resulting in endothelial dysfunction, platelet aggregation, and vascular thrombosis. While other xenoantigens, such as N-glycolylneuraminic acid and the SD(a) antigen, may also contribute to the xenogeneic immune response, their impact is generally considered secondary to the potent immunogenicity of αGal.^6^^,^^9^^,^^26^ Notably, transcatheter aortic valve implantation, similar to conventional surgical valve replacement, has been shown to induce the production of αGal-specific IgG3.^8^ Currently, all commercially available BHVs continue to express various xenoantigens, making it difficult to establish a definitive threshold below which antigen exposure fails to elicit a measurable immune response in the host.

Recent advances have further clarified the immunological mechanisms underlying intolerance to the αGal antigen, particularly its involvement in the pathogenesis of meat-induced allergic reactions. Individuals affected by this condition (referred to as “alpha-gal syndrome”) experience delayed anaphylactic responses following the ingestion of red meat products such as beef, pork, or lamb.^30^ Sensitization to αGal, however, is not limited to dietary exposure. A wide range of animal-derived pharmaceutical agents, including gelatin, heparin, Gelofusine, antivenoms, vaccines, monoclonal Abs, magnesium stearate, collagen-based drug capsules, and conjugated estrogens, have also been implicated in eliciting hypersensitivity reactions.^9^ This immunological phenomenon holds particular relevance in the context of BHVs, which are routinely manufactured from bovine or porcine tissues known to express high levels of αGal. In patients previously sensitized to αGal (either through food or pharmaceutical exposure), the implantation of such biomaterials may provoke an exacerbated immune response. This, in turn, could accelerate the structural deterioration of the prosthesis, as documented by Hawkins and colleagues.^10^ These observations underscore the importance of considering patient-specific immunological histories when selecting xenogeneic biomaterials for clinical use, particularly in cardiovascular applications.

The findings of this study confirm what has been preliminarily suggested in the literature, namely, that several commercially available BHV models retain a substantial burden of αGal antigens,^16^ despite having undergone various proprietary chemical treatments intended to reduce their immunogenicity.^3^, ^4^, ^5^, ^6^ A distinguishing feature of the present investigation lies in its systematic quantification of αGal antigen content for each BHV model, as detailed in Table 1 and Figure 3. When comparing the present results with previously published data,^16^ an approximately one order of magnitude increase in detectable αGal epitopes can be observed. This discrepancy is not indicative of a true biological increase but rather reflects the significant improvements made to the ELISA protocol used in this study, which has led to enhanced sensitivity and specificity. Notable methodological improvements include optimized sample incubation buffers to enhance Ab–antigen binding, the use of αGal–HSA (instead of αGal–BSA) to reduce nonspecific background, and an improved dilution buffer for the M86 monoclonal Ab.

Among the examined devices, the Trifecta GT valve (recently withdrawn from the market due to a high incidence of SVD) was found to exhibit the lowest number of αGal epitopes. Interestingly, the predominant mechanism underlying the clinical failure of the Trifecta GT does not appear to be primarily immune-mediated. Instead, evidence suggests that mechanical deterioration, particularly in the commissural regions, plays a central role. Repetitive leaflet-stent contact in these areas is believed to result in leaflet tearing and fibrotic remodeling, even in the absence of significant calcification.^31^, ^32^, ^33^ This observation highlights the considerable advancements achieved in the structural design of contemporary BHVs and underscores the need to further focus on strategies aimed at preserving the integrity of the biological tissue component. Enhancing tissue durability remains a critical objective in the pursuit of longer-lasting and more biocompatible heart valve prostheses.

While the exact threshold of αGal epitope exposure required to elicit a clinically relevant immune response is not yet fully defined, an implanted commercial BHV fixed in GA already exposes a significant number of αGal epitopes demonstrated to be sufficient to stimulate a measurable humoral immune response, including increased anti-αGal Ab titers.^11^ Therefore, additional exposure of epitopes due to GA depolymerization cannot be considered the cause of a new immune response; however, it certainly plays a crucial role in sustaining the inflammatory reaction over time. As described by Böer and colleagues,^11^ anti-αGal Ab levels remain elevated for up to 5 years following BHV implantation, suggesting a persistent inflammatory stimulus. These findings support the hypothesis that prolonged immunological stimulation may be attributable, at least in part, to the chemical instability of GA over time, which progressively reveals previously masked αGal epitopes,^33^ thereby contributing to the maintenance of the perception of the prosthesis as a foreign body. In the present study, a gradual decline in GA crosslinking efficacy was observed, coinciding with a 60% increase in exposed αGal epitopes 9 years after the initial fixation process (Figure 4).

It is important to acknowledge that the in vitro model employed for this analysis did not replicate the full spectrum of mechanical and fluid-dynamic stresses encountered by BHVs in vivo. These additional stressors could potentially accelerate the degradation of GA crosslinks, thereby amplifying the unmasking of immunogenic epitopes. Consequently, while the observed phenomenon provides compelling preliminary evidence, the interpretation of αGal re-exposure remains speculative at this stage. Future investigations utilizing αGal knockout large animal models may offer more definitive insights into the long-term immunogenic behavior of GA-fixed xenogeneic tissues under physiological conditions.

A second notable finding of this investigation concerns the efficacy of an experimental polyphenol-based treatment, which demonstrated the capacity to make inactive up to 99% of αGal antigens across all tested BHV models. Prior studies have indicated that polyphenols interact with GA-fixed tissues by forming a three-dimensional network that physically conceals reactive molecular sites within the extracellular matrix, including immunogenic xenoantigens.^17^, ^18^, ^19^ Spectroscopic analyses, particularly ^13^C nuclear magnetic resonance, have elucidated the nature of these interactions, revealing the formation of multiple types of chemical bonds (covalent and hydrogen bonds, π-π stacking interactions, and Van der Waals forces) that facilitate the stable incorporation of polyphenols into the tissue architecture.^16^^,^^25^^,^^29^^,^^34^ Beyond their capacity to neutralize antigenic determinants, polyphenols appear to exert a broader protective effect on the structural integrity of bioprosthetic tissues. In particular, they have been shown to mitigate several key processes associated with BHV degradation, including microbial colonization, the initiation of calcium crystal nucleation, and platelet adhesion.^16^^,^^35^ Moreover, the potential use of polyphenols in the treatment of BHVs is supported by extensive toxicological data and clinical evidence, which indicate a favorable safety profile at concentrations relevant to tissue processing.^36^, ^37^, ^38^, ^39^, ^40^, ^41^ While the exact quantity of polyphenols covalently bound to the tissue remains difficult to determine, only a small fraction of the initial dose is retained in the biomaterial (less than 500 μg/cm^3^), supporting a low risk of systemic toxicity. In addition, previous studies have demonstrated the long-term durability of polyphenol-based treatments on xenogeneic tissues and the stability of the bound compounds over time.^42^ These multifaceted properties highlight the therapeutic potential of polyphenols as a tissue-stabilizing agent, supporting their further investigation as a strategy to enhance the durability and biocompatibility of BHVs. Finally, optimizing the ELISA protocol was a critical advancement in this study, enabling more accurate detection of residual αGal epitopes in BHV tissues. Earlier assays, as in previous work,^16^ likely underestimated the true immunogenic burden due to limited sensitivity. Such underestimation may obscure the risk of chronic immune activation, contributing to early valve degeneration. The refined methodology enhances the reliability of xenoantigen quantification, facilitating a more accurate evaluation of BHV safety and informing the development of next-generation valves with improved biocompatibility and durability.

In conclusion, the findings of this study demonstrate that all evaluated BHVs retain measurable levels of αGal epitopes capable of eliciting an immune response. This persistent xenoantigenicity highlights a fundamental challenge in the design and processing of BHVs. While SVD is well recognized as a multifactorial process, influenced by mechanical stress, lipid infiltration, and the progressive instability of GA crosslinking, the role of immune-mediated mechanisms, particularly those linked to αGal antigenicity, has become increasingly evident as a critical driver in both the initiation and progression of calcific degeneration.^3^, ^4^, ^5^, ^6^^,^^9^^,^^12^, ^13^, ^14^ The immune response to αGal acts as a molecular trigger that sets in motion a complex cascade involving both the innate and adaptive immune systems. Such interplay underscores that αGal reactivity is not an isolated phenomenon but rather part of an intricate network of biological events that collectively compromise valve durability. Moreover, while current data support the effectiveness of polyphenols in masking the αGal epitope, the duration of antigen inactivation under in vivo conditions remains under investigation. Accordingly, ongoing investigations using ovine models are planned to validate the translational relevance of these findings and to assess the long-term functional performance and immunological profile of polyphenol-treated xenogeneic tissues under dynamic biomechanical conditions.

## Ethics Statement

This research was conducted in accordance with the ethical standards and guidelines applicable to scientific research and publication.

## Funding

This work was supported by Biocompatibility Innovation Srl (Este, Padua, Italy).

## Review Statement

The review of this manuscript was managed by Guest Editor Stephanie Sellers, PhD.

## Disclosure Statement

I. George and A. Colli are clinical advisors for Biocompatibility Innovation. F. Naso is Biocompatibility Innovation's Chief Technology and Innovation Officer. The other authors had no conflicts to declare. Biocompatibility Innovation (BCI) is a company dedicated to developing technologies that enhance the biocompatibility and durability of implantable biomedical devices (IMDs). BCI does not manufacture any IMDs, nor does it operate production facilities. The polyphenol-based technology described in the manuscript is still in the experimental phase and is currently being evaluated for its scientific and translational potential. At present, it has no commercial application, and it remains uncertain whether it will ever reach the stage of clinical or industrial deployment.
